# Supplementary material for: Study on the failure of oil-contaminated wheel-rail conditions
Source: PLoS One. 2025 Mar 12;20(3):e0318257. doi: 10.1371/journal.pone.0318257 (PMC11902288; doi:10.1371/journal.pone.0318257)
Supplement: Attachment — Submitted filename: Response to Reviewers.docx [file pone.0318257.s002.docx]

**Response to Reviewers**

**Dear reviewers:**

Thank you for your decision and constructive comments on my manuscript. The red part that has been revised according to your comments in paper. Revision notes, point-to-point, are given as follows:

**Reviewer #1:**

**This manuscript set out to investigate whether oil exhibits a similar adhesion improvement phenomenon as observed under water conditions, for which a wheel-rail adhesion test was conducted. The results prove that adhesion improvement can occur on oil-contaminated rail surfaces when there is greater wheel sliding. Finally, the study analyses the failure mechanism and influencing factors of the oil-contaminated interface, proposes the conditions under which failure occurs. The topic of the manuscript is novel and puts forward a new point of view. It could be accepted for publication after minor revision. The relevant comments are listed below.**

(1) **Since the circumferential test rig is used, the experiment reflects the operation of the wheels on the curved track. Under the condition of oil in the linear track, are the conditions for adhesion improvement consistent?**

Thank you for your suggestions. Relevant explanations have been added to Section 2.1. In fact, considerations regarding the influence of curves and straight sections were incorporated from the initial design phase of the test rig. This test rig employs a circular track with a diameter of 2 meters, and the wheel-rail contact surface is illustrated in Figure 1. The impact of the curve lies in the slight difference in linear velocity between the inner and outer sides of the wheel-rail contact patch, resulting in a spin moment within the contact patch. The magnitude of this moment is influenced by the diameter of the circular track^[1]^.

Figure 1 Spin torque between wheel and rail

Based on preliminary calculations^[1]^, it was determined that the torque generated by a track with a 2-meter diameter differs by less than 5% compared to a straight track, which meets engineering requirements, as shown in Figure 2. Furthermore, since this test is primarily conducted under conditions where slip ratio reaches up to 80%, full sliding occurs within the wheel contact patch. Under such conditions, there is minimal difference in adhesion characteristics between straight and curved tracks.

Figure 2 Research Results

(2) **When the circumferential test rig is running at high speeds for long periods of time, the rail also accumulate heat, does this have an impact on the test results?**

Thank you for the suggestions. I believe that the impact of rail temperature can be reasonably neglected. After halting the test, I manually touched both the rail and wheel surfaces, finding that the rail surface showed almost no temperature increase, while the wheel was noticeably hot. This is likely due to the cyclic rolling and sliding of the wheel, which continuously absorbs heat on its surface, making it difficult for the heat to dissipate. In contrast, the circular track has a larger surface area for heat dissipation, preventing significant heat accumulation. Therefore, it seems reasonable to disregard rail temperature increases in this context. Unfortunately, I did not measure the rail surface temperature, so data on rail temperature cannot be included in this paper.

**(3) In experimental research on wheel-rail adhesion under clean and dry conditions, Why is the target slip rate set to a sinusoidal curve? What is the slip rate law of the previous experiment? Please indicate this in the text.**

Thank you for the suggestion. Additional references on the target slip ratio settings in other studies have been added in Section 2.2. Setting the slip ratio as a sine wave facilitates easier motor control. When measuring wheel-rail adhesion, achieving a significant wheel sliding is sufficient to capture the adhesion at that moment. However, if the slip rate changes too rapidly, the data collected can become highly scattered. Using a sine wave as the target slip ratio curve provides a continuously differentiable function, allowing for more precise adhesion data collection.

(3) **From Figure 7, as the speed increases, the maximum wheel rail adhesion coefficient decreases, and the corresponding slip ratio increases. Please explain the reason in detail. Suggest analyzing the correlation between speed and wheel rail force, and then analyzing the characteristics of Figure 7.**

Thank you for your suggestion. Additional explanations have been incorporated into Section 3.4. It is generally accepted that the adhesion coefficient between the wheel and rail is influenced by speed, with higher speeds typically resulting in lower adhesion coefficients. Previous studies^[2]-[5]^ have extensively demonstrated the inverse relationship between speed and wheel-rail forces. In this study, tests were conducted on a dry rail surface to establish the adhesion characteristics under dry conditions, providing a control group for comparison with low-adhesion conditions using an oil medium.

**Reviewer #2:**

**The authors utilized a circumferential test rig to construct oil-contaminated wheel-rail contact interfaces. Adhesion characteristic tests were conducted over a slip ratio range of up to 80% to determine whether adhesion improvement phenomena can occur with high-sliding under oil conditions. Overall, this is an excellent study. However, the following question should be addressed.**

(1) **Please ensure that your manuscript meets PLOS ONE's style requirements, including those for figure caption and references.**

Thank you for your suggestion. I have revised the formatting throughout the paper and enhanced the clarity of all figures.

(2) **Compare the results obtained in this study with other experiments or studies in literature. Although you have mentioned ‘In previous literature, the relationship between the adhesion coefficient and slip ratio typically shows a declining trend after reaching a saturation point, and the adhesion coefficient-slip ratio curve for oil-contaminated wheel-rail interfaces is a single-peaked curve.’ in the manuscript, it would be helpful if deeper and quantitative analysis could be provided.**

Thank you for your suggestion. Indeed, tests under oil conditions have rarely been conducted at such a high slip ratio of 80%, as it was previously considered unnecessary. I did not add a comparison with previous literature results in the main text because existing studies[6]-[8] primarily report data from tests conducted under constant slip conditions, often with single trials. These results are difficult to directly compare in a single figure with the continuous testing performed in this study.

Figure 3 Experimental results in reference^[6]^

(3) **The main findings of the paper need to be mentioned in the abstract.**

Thank you for your suggestion. I have revised the abstract to highlight the study's findings under oil condition.

(4) **The figures are of poor quality. Could the authors please improve its resolution?**

Thank you for your suggestion. I have reformatted the document and improved the figure quality.

(5) **Apart from experimental study, could the authors supplement numerical study of the same oil-contaminated wheel-rail conditions to prove the experimental study results?**

Thank you for your suggestion. Although I have conducted some numerical studies, it is not suitable to present them in this paper, as the focus is primarily on experimental research in this paper. However, a few selected numerical examples can be presented here. The numerical method used in this study is based on the mixed lubrication theory in bearing lubrication[6]. Using the unified Reynolds equation, a mixed lubrication wheel-rail contact model under oil conditions was developed. Figure 4 below illustrates the contact characteristics under conditions of 80 km/h, 14t axle load, and 80% slip ratio, including metal roughness peak contact, oil film contact, and the oil film temperature rise at the wheel-rail interface.

Figure 4 Numerical Calculation Results

The numerical method is relatively complex and is not suitable for presentation in this paper. Currently, the method is only applicable for short-duration simulations, as simulating for several tens of seconds requires a prohibitively large computational effort. For long-duration simulations, simplified analytical solutions would need to be explored, which presents a new challenge.

**Reviewer #3:**

**Based on these assessments, I recommend accepting the manuscript, with minor proofreading and formatting adjustments to enhance its final presentation quality before publication. While the manuscript is technically sound, there are a few minor suggestions that could enhance clarity and precision. Below are specific recommendations for refinement:**

(1) **There is an error in the notation used in Equation 1.**

Thank you for your suggestion. I have corrected the error in Equation 1.

(2) **The manuscript describes manual application of oil (type 75W-90) on the rail surface. Should the effect of oil film thickness on the adhesion coefficient be considered?**

Thank you for your suggestion. I have added some clarifications in Section 2.2 of the paper. The oil film thickness is indeed an important parameter, but under non-starved conditions, excess oil cannot enter the wheel-rail contact interface. We ensure sufficient lubrication and avoid starved conditions by controlling the amount of oil applied to the rail surface. While the oil film thickness between the wheel and rail is difficult to measure, we maintain consistent experimental results by supplying excess oil, ensuring that the lubrication conditions are similar for each test.

(3) **For Figure 8, could approximate data ranges be provided for the S1, S2, and S3 data points?**

Thank you for your suggestion. Figure 8 is merely a schematic diagram. As shown in Figures 4-6 of the paper, due to the thermal accumulation on the wheel surface caused by continuous sliding, the slip ratio and adhesion coefficient values at points S1, S2, and S3 are different. In fact, during the continuous sliding of the wheel, the adhesion coefficient dynamically changes. Therefore, it is difficult to provide a range for the slip ratio and adhesion coefficient at points S1, S2, and S3 in Figure 8.

(4) **In Figure 9, the temperature distribution for the wheel surface is shown. Is the temperature data based on the average temperature of the wheel surface?**

Thank you for your question. I have enlarged Figure 9 and added relevant explanations in Section 4.1 of the paper. The temperature data presented is based on the average temperature within the selected contact patch.

(5) **It is recommended that all figures undergo further refinement to enhance clarity. To ensure your figures meet our technical requirements, please run each figure included in your submission files through the PACE tool: https://pacev2.apexcovantage.com/.**

Thank you for your suggestion. I have reformatted the entire paper and improved the clarity of the figures.

**References**

[1] Chao Chen, Chun Tian, Jiajun Zhou, et al, Development of a New Test-Rigfor Wheel- Rail Contact Experiments under Large Slip Conditions, Applied Sciences. 13(9) (2023) 5320
[2] Zhou jiajun, Wu Mengling, Tian Chun, et al, Experimental investigation on wheel–rail adhesion characteristics under water and large sliding conditions, Ind Lubr Tribol. 73(2) (2020) 366-372.
[3] Chang Chongyi, Chen Bo, Cai Yuanwu, et al, Experimental Study on Large Creepage Adhesion of Wheel/Rail Braking at 400 km·h-1 (Ⅰ)—Adhesion Characteristics under Water Medium, China Railway Science. 43(2) (2022) 96-103. In Chinese.
[4] Chang Chongyi, Chen Bo, Cai Yuanwu, et al. Experimental Study on Large Creepage Adhesion of Wheel/Rail Braking at 400 km·h-1(II), China Railway Science. 43(05) (2022) 87-93. In Chinese.
[5] Chongyi Chang, Bo Chen, Yuanwu Cai, et al, Experimental investigation of high-speed wheel-rail adhesion characteristics under large creepage and water conditions, Wear. 540-541 (2024) 205254.
[6] E.A. Gallardo-Hernandez, R. Lewis, Twin disk assessment of wheel/rail adhe sion, Wear 265 (9–10) (2008) 1309–1316.
[7] Gallardo-Hernandez EA, Lewis R, Twin disc assessment of wheel/rail adhesion, Wear. 265(9) (2008) 1309-1316.
[8] Grieve DG, Lewis R, Gao N, Wear and fatigue of railway track caused by contamination, sanding and surface damage, Acta Orthop Belg. 2003, 39(6) (2003) 1007-1015.
[9] Hu Yuanzhong , Zhu Dong . A Full Numerical Solution to the Mixed Lubrication in Point Contacts[J]. Journal of Tribology, 2000, 122(1).
